# Nanoplasmonics Enabling Cancer Diagnostics and Therapy

**DOI:** 10.3390/cancers14235737

**Published:** 2022-11-22

**Authors:** Ren A. Odion, Yang Liu, Tuan Vo-Dinh

**Affiliations:** 1 Fitzpatrick Institute for Photonics, Duke University, Durham, NC 27708, USA; 2 Department of Biomedical Engineering, Duke University, Durham, NC 27708, USA; 3 Department of Chemistry, Duke University, Durham, NC 27708, USA

**Keywords:** plasmonics, gold nanostar, photothermal, immunotherapy

## Abstract

**Simple Summary:**

Recent advances in the state of the art for early cancer detection and therapy have led to a paradigm shift in the way the disease is detected and treated. In particular, treatments have become much more targeted and localized to minimize systemic body-wide side effects common in traditional treatment methods such as chemotherapy. At the forefront of this revolution is the utilization of plasmonic gold nanoparticles, which have gained increasing attention as a highly effective nanoplatform ranging from drug delivery to plasmonics-enhanced treatments such as photothermal therapy.

**Abstract:**

In this paper, we highlight several advances our laboratory has developed in the pursuit of cancer diagnostics and therapeutics by integrating plasmonics, photonics, and nanotechnology. We discuss the development and applications of plasmonics-active gold nanostar (GNS), a uniquely shaped nanoparticle with numerous branches that serve to greatly amplify the thermal generation at resonant wavelengths. GNS has also been successfully used in tumor imaging contexts from two-photon fluorescence to surface-enhanced Raman scattering (SERS) sensing and imaging. Finally, GNS has been coupled with immunotherapy applications to serve as an effective adjuvant to immune checkpoint inhibitors. This combination of GNS and immunotherapy, the so called synergistic immuno photo nanotherapy (SYMPHONY), has been shown to be effective at controlling long-lasting cancer immunity and metastatic tumors.

## 1. Introduction

Cancer has become one of the most deadly diseases throughout the world, with nearly 2 million new cases and over half a million deaths in the U.S. in 2022 alone [1,2]. Of the more deadly cancers, glioblastoma (GBM), is an especially devastating cancer of the brain [3,4]. GBM patient survival rate is a rather dismal at 15 months, even after treatments such as surgery, chemotherapy, and X-ray radiotherapy (XRT) [3,4]. Despite enormous efforts, GBM is still a deadly disease with essentially 100% mortality, urging for new treatment methods. Cancer detection has traditionally relied on techniques that can penetrate thick tissue and produce images that delineate non-tumor tissue from normal tissue based on its structure and density. Modalities such as computed tomography (CT) and magnetic resonance imaging (MRI) are particularly effective in delineating tumor masses from normal tissue, as cancer tissue often has an abnormal tissue density compared with its local environment [5,6]. However, these modalities are often limited as they require highly trained doctors and technicians who can interpret CT and MRI data to make a diagnosis. Additionally, these methods require further interrogations of suspected tumors via histopathology to ascertain the presence of cancerous growth [7].

To overcome these issues, optical modalities that probe the biomolecular composition of the tissue itself have been developed to non-invasively diagnose cancer. Methods such as absorption, fluorescence, and Raman spectroscopy are all modalities that can probe for the unique biochemical composition of cancer tissue, allowing for in vivo imaging that provides a high molecular specificity [8,9,10,11,12,13,14]. The absorption and scattering optical properties are distinct in different tissues and serve as the contrasting property of modalities such as diffuse optical tomography (DOT) [9,15]. On the other hand, fluorescence arises from the sum of different metabolic fluorescence molecules, which can serve as an effective indicator for tumor tissues that have abnormal metabolic rates [10]. Raman spectroscopy is the most specific as the spectral fingerprint of a molecule will have unique Raman peaks at specific Raman shift values.

All of these methods can be utilized with minimal equipment and training, and thus could be useful for rapid diagnostic imaging. However, intrinsic signal differences between tumor and non-tumor tissue can be noisy. In the case of Raman spectroscopy, other sources of signals such as fluorescence may overwhelm the weak Raman scattering signal [16]. One way to overcome this limitation is to use a fluorescent or Raman dye targeted to the tumor [17,18,19]. This can be achieved with methods such as antibody targeting, but recently, nanoparticles have become an ideal choice as a delivery platform as they can take advantage of the enhanced permeation and retention (EPR) effect in which the leaky vasculature of tumor sites allows for nanoparticle accumulation to the local tumor site [17,20]. Additionally, the use of gold, which is a biocompatible material, allows for flexible chemical conjugation of drugs or dyes into these particles, allowing for multifunctional uses of detection and treatment [18,21,22]. Gold nanoparticles, such as gold nanoshells, have been used for photothermal ablation of prostate tumors in clinical trials with no significant toxicities [23]. In addition, a first-in-human clinical study of RNA interference-based spherical nucleic acids, which consist of gold nanoparticle cores, was conducted in patients with recurrent glioblastoma and it revealed no grade 4 or 5 treatment-related toxicities [24]. Finally, these metallic nanoparticles have a unique absorption signature that makes them ideal for targeted thermal therapy [20,25,26].

Here, we provide an overview of the development of the gold nanostar (GNS) and its applications as a tumor imaging nanoplatform, ranging from its use as a diagnostic tool to a highly specific tumor treatment modality.

### 1.1. Plasmonic Nanoparticles for Tumor Imaging and Treatment

**Figure 1:**
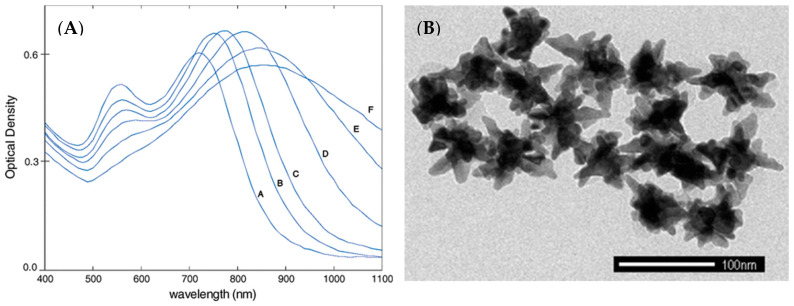
(**A**) Absorption tunability of different sized gold nanostars with letters A–F corresponding to nanostar sizes 45 nm, 52 nm, 57 nm, 72 nm, 94 nm, and 116 nm, respectively. (**B**) TEM image of gold nanostar (adapted from Ref. [35]).

Plasmonic-active nanosystems such as metallic nanostructures and nanoparticles have recently seen a wide usage of applications ranging from diagnostics to therapy. Plasmonics is a field associated with the induced oscillation of conduction electrons found in metallic nanoscale structures using a source such as laser light. These electron oscillations, or surface plasmons, produce a secondary electromagnetic field that augments the incident electromagnetic field, resulting in a large electromagnetic field around the nanostructure. Numerous plasmonic-active nanoparticles of different geometric shapes and metallic compositions have been developed for tumor imaging. Gold nanospheres have also been used and have seen applications ranging from photothermal generation to drug delivery [27,28,29]. They have also been used as the core to larger nanoparticle constructs used in multimodal applications such as in ultrasound and photoacoustic imaging coupled with fluorescence [30,31]. Other particles such as the gold nanoshell are specifically tuned to near IR absorption to allow for reaching maximal in-depth tissue for cancer therapy [23,27,32,33,34]. Among the nanoparticles with different morphologies and shapes, GNS is among the most effective nanoplatforms for its unique star-shaped geometry and optical tunability [35,36,37]. The plasmonic E-field enhancement is concentrated around the tips of each star, which act as a “lightning rod” that greatly exceeds the enhancement of smoother particles such as nanospheres [38,39]. This electromagnetic (EM) field greatly increases Raman scattering near the surface of the metal in what is known as surface-enhanced Raman scattering (SERS), allowing for chemical species adsorbed on or near the surface of the metal to have a very strong Raman signal. Our laboratory has developed a variety of metallic nanoparticles for SERS applications in chemical analysis, sensing, and diagnostics [40,41,42,43]. This EM field enhancement around the nanoparticle manifests in a highly absorbing nanoparticle that has a high photon-to-heat conversion [17]. Figure 1A shows the GNS particle’s flexibility in absorption across different wavelengths, allowing for possible tuning of the precise excitation laser for targeted photothermal therapy, which minimizes off-target heating, while Figure 1B shows the transmission electron microscopy (TEM) image of the GNS nanoparticles [35]. The two-photon luminescence (TPL) of GNS is also greatly enhanced, which allows for label-free imaging and tracking of nanoparticles in the vasculature [18,20,44]. Such a technique allows for GNS to act as a contrast agent, which demonstrates the extravasation of nanostars in the brain. 

**Figure 2:**
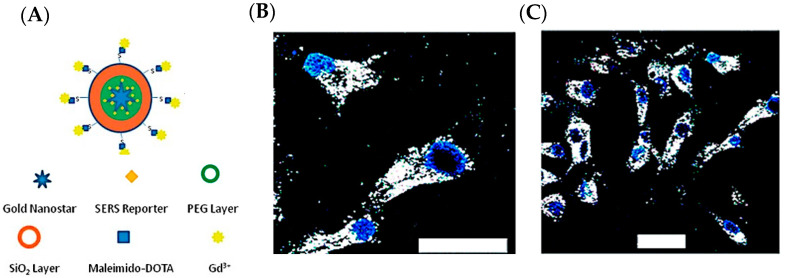
(**A**) Multifunctional gold nanostar. (**B**,**C**) Two-photon luminescent images of nanoprobes incubated within BT549 cancer cells after 4 h and 14 h, respectively. The nucleus is stained blue, and the particles are shown as white. Scale bar is at 50 μm (adapted from Ref. [18]).

As a demonstration of theranostics, we developed a specially designed multifunctional gold nanostar with multiple functionalities in detection (SERS, MRI, CT, and TPL) and photothermal treatment (PTT), allowing for pre-operative macroscopic imaging as well as photothermal therapy for post-operative treatment [18]. Furthermore, our group has specifically developed a surfactant-free fabrication of GNS unlike many other similar gold nanoparticle synthesis methods [37,39]. This allows for an even greater biocompatibility for in vivo applications. Additionally, the surface can easily be functionalized with a variety of other reporters for imaging modalities such as MRI [18]. Figure 2A shows the different possible functionalization and coating options for GNS. Figure 2B,C demonstrates the ability for this particle to accumulate within cancer cells (BT-549). 

**Figure 3:**
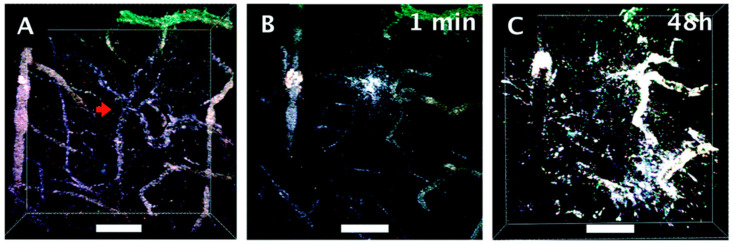
(**A**–**C**) TPL images of the blood–brain barrier through a cranial window. Scale bar is 100 μm. The red arrow indicates the vasculature tortuosity in the tissue. (**A**) Tumor vessels prior to heating, (**B**) 1 min after heating, and (**C**) 48 h after heating. Increased presence of GNS (colored white) found in significant quantities around blood vessels after 48 h (adapted from Ref. [44]).

**Figure 4:**
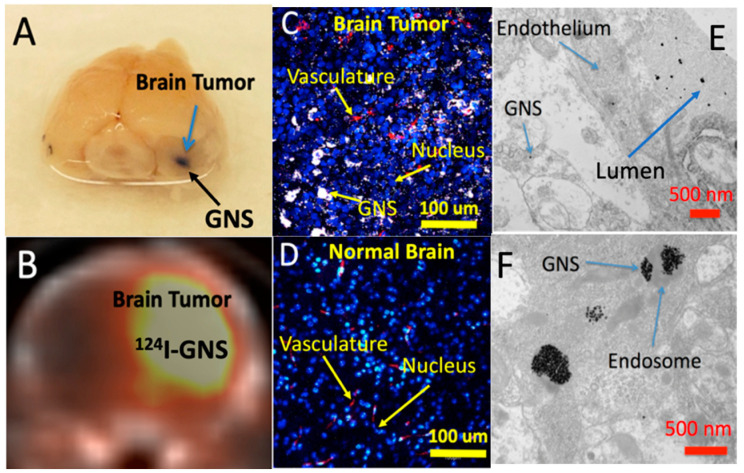
GNS selective accumulation in GBM after intravenous (IV) injection. (**A**) Mouse brain sample image shows light-absorbing GNS in the brain tumor 24 h after IV injection. (**B**) PET/CT shows 124I-GNS accumulation in the brain tumor 48 h after IV injection. Two-photon microscopy shows GNS (white) only in the brain tumor (**C**), but not the healthy brain (**D**). Blue: nucleus. Red: blood vasculature. Electron microscopy shows that GNS penetrated through brain tumor vasculature (**E**) and were found inside the GBM cell (**F**) after IV injection (adapted from Ref. [19]).

We have also demonstrated that GNS can accumulate selectively in brain tumors through a disrupted blood–brain barrier (BBB) using intracranial GBM murine animal models [19,44]. As shown in Figure 3, GNS is localized around the surrounding tumor interstitial space, vasculature, and lumen, suggesting that GNS nanoparticles penetrated through the GBM vasculature following laser excitation [44]. From this work, we also demonstrated that hyperthermia (HT) generated from GNS-enhanced photothermal treatment can increase GBM vasculature permeability to improve drug delivery into the tumor. Hyperthermia, a treatment whereby heat is applied to a tumor to increase the temperature above the physiologic body temperature (~37 °C), can dramatically enhance BBB permeability, allowing for the passage of large molecules and immune cells into heated tissues. Laser-induced HT can increase GNS nanoparticles’ extravasation into GBM by using TPL imaging and a murine animal model (Figure 3) [44]. Figure 4A shows GNS with black accumulated selectively in the brain tumor 24 h after intravenous (IV) injection. We performed a PET/CT scan with ^124^I-labeled GNS, and the measured brain tumor uptake was up to 7.2% ID/g 48 h after intravenous (IV) injection (Figure 4B). Two-photon photoluminescence (TPL) imaging confirmed that GNS accumulated selectively in the brain tumor, but not in the surrounding healthy brain tissue after IV injection (Figure 4C,D). In addition, we investigated the GNS’s subcellular location in GBM by using electron microscopy imaging. The experimental results showed that the vasculature of GBM was disrupted and permeable for GNS nanoparticles with a size of 50 nm. GNS can be identified in the lumen, blood vasculature, and tumor interstitial space, indicating that GNS nanoparticles leaked through the vasculature in GBM (Figure 4E). GNS nanoparticles were also found in the intracellular vesicles within the GBM cell (Figure 4F). 

In recent years, there has been growing interest in the development of various approaches of immunotherapy for cancer treatment. PTT-based treatment for cancer using GNS effectively induces HT in localized areas where nanoparticles have accumulated (such as around tumors). However, local treatment will potentially miss distant and not yet found tumors, which may result in cancer remission. As such, PTT can be improved with the introduction of immunotherapy as a combinatorial agent for better cancer therapy effectiveness [26]. Immune checkpoint inhibitors have recently emerged as a promising cancer treatment. Proteins such as Programmed Death Ligand 1 (PD-L1) are targeted in immunotherapies as they are responsible for the suppression of the immune system, notably activated T cells, B cells, and myeloid cells [26,45,46,47,48]. The therapeutic anti-PD-L1 antibody blocks this interaction with PD-L1 and its receptor PD-1 found on the surface of immune cells, allowing for the activation of normal immune functions. Subsequently, this has led to the promising benefit of long-term recognition of tumors by T cells, which will inevitably allow for the treatment of distant and metastatic tumors. To further augment this treatment modality, we combined GNS-enabled PTT with immunotherapy in order to develop a novel synergistic therapy. This two-stage treatment is referred to as Synergistic Immuno Photo Nanotherapy (SYMPHONY) [26,49]. The first step in this treatment involved localized PTT with NIR irradiation on GNS, which were absorbed into tumors through EPR. This results in the thermal destruction of primary tumors. After this, antigen-presenting cells (APCs) can record released tumor antigens including damage-associated molecular pattern molecules (DAMPs) and heat shock proteins (HSPs) [50,51,52]. These APCs inevitably process these tumor antigens and present them to T cells, which will attack primary and distant cancer cells. The combination of GNS-PTT and immunotherapy acts in a synergistic manner that ultimately results in long-term cancer cell recognition and metastatic tumor treatment.

**Figure 5:**
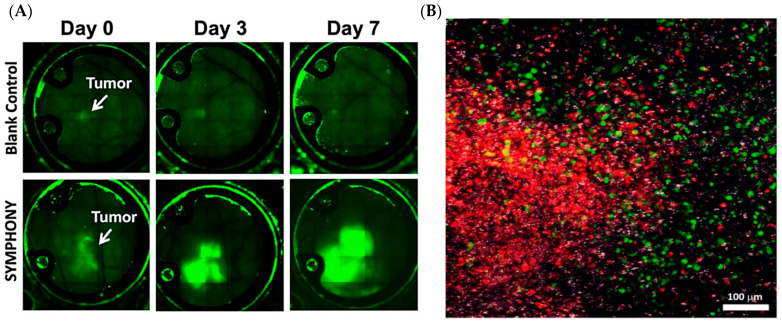
Intravital optical imaging of the immune response through the window chamber using CX3CR1-GFP mice with the EGFP expression in immune cells. (**A**) 10× magnification fluorescence images showing the accumulation of immune cells in the tumor under SYMPHONY photoimmunotherapy. (**B**) TPL images showing the interaction between tumor cells (red), immune cells (green), and GNS (white) in the distant tumor region (adapted from Ref. [25]).

We have continued this work on the study of in vivo interactions with nanoparticles through intravital optical imaging through a dorsal skinfold window chamber animal model [25]. Previously, we have shown immune cell accumulation within distant tumors after the treatment of only the primary tumor, demonstrating the potential for an anti-cancer vaccine-like effect where distant/metastatic cancer can be effectively treated. This treatment scheme can be modelled using a dual flank tumor mouse model, where only one flank is treated with PTT and then subsequently treated with anti-PD-L1 [26]. The other flank tumor thus acts as the distant metastatic tumor model that is not photothermally treated but is effectively eradicated by the resulting immune response following SYMPHONY. Of particular note, neither PTT nor immunotherapy alone have shown effective long lasting cancer withdrawal in these dual flank models, indicating that it is only through the synergistic combination of GNS PTT and immunotherapy that a long-term vaccine-like effect to cancer treatment can be achieved [26]. Figure 5A shows fluorescence imaging that was used to demonstrate that stained immune cells selectively accumulated in the untreated non-primary tumor of a dual flank tumor mouse model after SYMPHONY treatment. Figure 5B shows a TPL image of the distant tumor site showing a wide mixture of tumor cells, immune cells (monocytes and macrophages), and GNS.

### 1.2. Photothermal Therapy (PTT)

The GNS nanoparticles’ multiple sharp branches result in tip-enhanced plasmonics for imaging and PTT [45,53,54]. Upon irradiation with a laser light source, the surface electrons on the metal are thrown into oscillations and induce a strong EM force field. These oscillating plasmons can be roughly modeled after the Drude model of electric conduction at the nanometer scale. Previously, our group developed several models with finite element software (COMSOL Multiphysics) to determine the bulk absorption profile across different wavelengths [38,45,54]. This is particularly important as optical tuning of GNS is possible by controlling the amount of gold or silver added in the synthesis of the particles, and the peak absorption can be red or blue shifted depending on the final size of the particles [35,37,39]. For example, adding more silver chloride reactant in the synthesis notably red-shifts the peak absorption [39]. To this end, we developed GNS targeted at the near-infrared (NIR) spectral range (700–1100 nm) within the so-called “tissue optical window”, which corresponds to the wavelength range of least absorption by the tissue [35,37,45].

**Figure 6:**
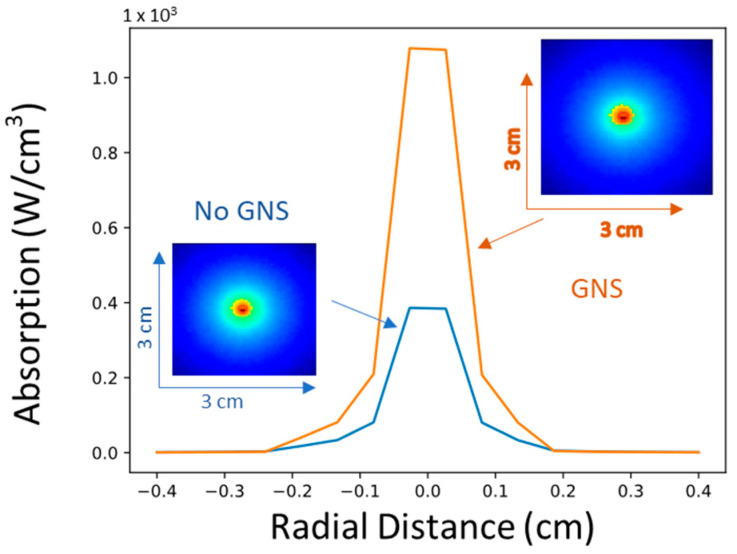
Monte Carlo photon propagation simulations for glioblastoma mimicking phantoms where GNS localized within a tumor volume of 0.5 cm radius under a point source excitation within the tumor. Absorption is significantly increased within the tumor volume with the addition of GNS in the simulation.

The thermal treatment of tumors can be achieved conventionally using microwave or high-intensity focused ultrasound. However, such methods are not suitable for deep-seated tumors and off-target heating is often a problem. To circumvent this issue, we utilize GNS to take advantage of the EPR effect and only allowed GNS to accumulate around the tumor tissue. As gold nanoparticles are relatively inert, off-target accumulation is not a concern and PTT will only occur in tissue directly irradiated by the laser. This NIR window is particularly important as targeting this range allows for maximal tissue penetration by the traveling photons and minimal absorption around the surrounding tissue. By using the accumulated GNS as the heat nanogenerators selectively absorbed inside tumors via the EPR effect, rather than heating the entire tissue itself, off-target heating can be greatly reduced. In Figure 6, we used a Monte Carlo Photon Propagation model to directly visualize a 2D slice of the absorption of tissue under specific excitation conditions [55]. We used the optical properties of gray matter in glioblastoma (u_a_ = 0.82 cm^−1^, u_s_ = 5.70 cm^−1^, g = 0.9) as the tissue model under 1064-nm excitation [56]. With the addition of GNS, an additional absorption sink is added into the Monte Carlo model and acts as new localized heat source, where we demonstrate the dramatic increase in photon absorption in the tumor tissue area. 

### 1.3. Synergistic Immuno Photo Nanotherapy (SYMPHONY)

**Figure 7:**
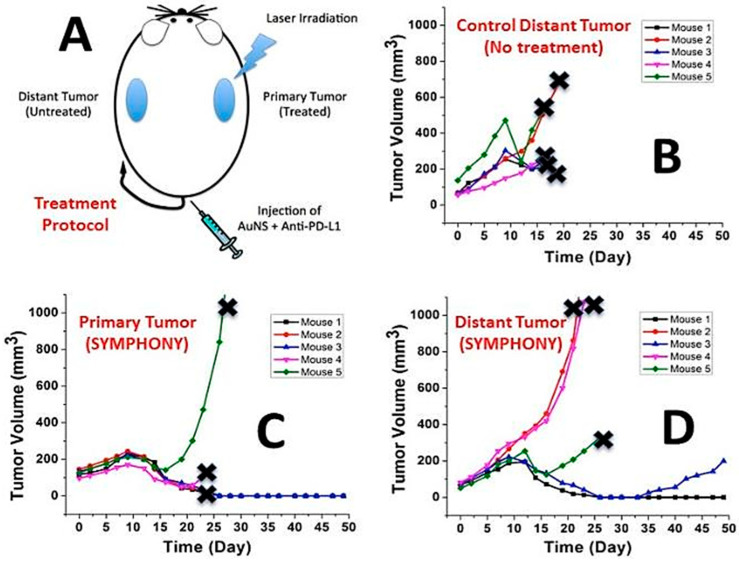
(**A**) Scheme of SYMPHONY photoimmunotherapy. (**B**) Tumor size change profile for mice in the control group without any treatment. (**C**) Primary tumor size change profile for mice in the SYMPHONY photoimmunotherapy group. (**D**) Distant tumor size change profile for mice in the SYMPHONY photoimmunotherapy group. The sign of x indicates that the mouse is sacrificed. (Adapted from Ref. [26]).

We used a bladder cancer murine animal model to demonstrate the proof of principle that a combination of photothermal therapy with immunotherapy could generate synergistic effects from the immune response triggering and tumor immune suppression reversion to treat not only primary tumors, but also a distant tumor, used as a model for cancer metastasis [26]. The principle of our SYMPHONY photoimmunotherapy is shown in Figure 7A. Our GNS nanoparticles can accumulate selectively in the tumor due to EPR. Moreover, the unique shape of GNS allows the nanoparticle to be highly efficient in converting NIR light to heat and generate local high temperatures enough to selectively kill cancer cells. In conjunction with the photothermal treatment, the anti-PD-L1 antibody is also administered. This immunotherapy antibody is a highly specific molecule that inhibits important molecular pathways that cancer cells use to suppress the immune response. As shown in Figure 7B, tumors in the control group without any treatment grew quickly and the mice died soon. For the mice with our SYMPHONY photoimmunotherapy, both the primary tumor with the laser treatment and the distant tumor without laser treatment had a significant growth inhibition (Figure 7C,D). The therapeutic efficacy of our SYMPHONY treatment was most readily seen from the 80% tumor response rate and 20% complete cure rate, indicating an effective long-term anti-cancer immune response. Preliminary murine studies have shown that our novel two-pronged therapeutic approach was effective at destroying the primary tumor where GNS-PTT was applied, as well as at distant untreated cancer sites. It also triggered an anti-tumor immune memory that prevented tumor growth in cured mice when they were re-challenged with a second injection of cancer cells (a vaccine effect not observed in PTT or immunotherapy alone).

## 2. Conclusions

Nanoplasmonics is a rapidly growing field for diagnostics and therapeutic applications. Theranostics applications using the multifunctional abilities of GNS can be used in a variety of ways ranging from non-invasive optical imaging to thermal treatment in combination with immunotherapy. GNS can also be selectively functionalized and loaded with different contrast agents for augmenting traditional cancer imaging techniques such as PET, MRI, and CT. This ability to detect cancer naturally leads to the GNS particle’s strength as a mediator for photothermal therapy, which can be selectively tuned to efficiently heat the target tumor tissue after taking advantage of the EPR effect. Finally, GNS can be used in conjunction with immunotherapy in the treatment of distant tumors by acting as an immunoadjuvant. Such an anticancer “vaccine” effect is possible, as distant tumors are effectively destroyed in SYMPHONY studies, wherein only one of the dual flank tumors on mice is treated. Future studies will look into this combinatorial effect between photothermal therapy and immunotherapy, with particular emphasis on the characterization of immune cells involved in the treatment of recurring and distant metastatic tumors.

## Data Availability

No new data were created or analyzed in this study. Data sharing is not applicable to this article.
